# Supplementary material for: Multi‐dataset identification of innovative feature genes and molecular mechanisms in keratoconus
Source: J Cell Mol Med. 2024 Sep 19;28(18):e70079. doi: 10.1111/jcmm.70079 (PMC11412914; doi:10.1111/jcmm.70079)
Supplement: Supplementary file 2 — Material S2. [file JCMM-28-e70079-s002.zip › 7.GSEA analysis/6.SNORA74B/KEGG_SYSTEMIC_LUPUS_ERYTHEMATOSUS.html]

Details for gene set KEGG\_SYSTEMIC\_LUPUS\_ERYTHEMATOSUS[GSEA]

|  || Dataset | SNORA74B.group.cls#h\_versus\_l |
| Phenotype | group.cls#h\_versus\_l |
| Upregulated in class | l |
| GeneSet | KEGG\_SYSTEMIC\_LUPUS\_ERYTHEMATOSUS |
| Enrichment Score (ES) | -0.5789379 |
| Normalized Enrichment Score (NES) | -1.4368216 |
| Nominal p-value | 0.077689245 |
| FDR q-value | 0.31217566 |
| FWER p-Value | 0.867 |
Table: GSEA Results Summary

  

Fig 1: Enrichment plot: KEGG\_SYSTEMIC\_LUPUS\_ERYTHEMATOSUS      
 Profile of the Running ES Score & Positions of GeneSet Members on the Rank Ordered List

  

| SYMBOL | TITLE | RANK IN GENE LIST | RANK METRIC SCORE | RUNNING ES | CORE ENRICHMENT || 1 | CD86 | na | 436 | 0.628 | 0.0374 | No |
| 2 | C9 | na | 2720 | 0.318 | -0.0031 | No |
| 3 | ELANE | na | 6174 | 0.187 | -0.0879 | No |
| 4 | C6 | na | 7702 | 0.143 | -0.1205 | No |
| 5 | FCGR1A | na | 8319 | 0.129 | -0.1280 | No |
| 6 | FCGR2A | na | 8572 | 0.123 | -0.1255 | No |
| 7 | GRIN2A | na | 8634 | 0.122 | -0.1176 | No |
| 8 | C8A | na | 8758 | 0.119 | -0.1117 | No |
| 9 | CD28 | na | 11178 | 0.072 | -0.1757 | No |
| 10 | HLA-DRB5 | na | 11281 | 0.070 | -0.1731 | No |
| 11 | ACTN4 | na | 12377 | 0.055 | -0.2003 | No |
| 12 | C8G | na | 13060 | 0.046 | -0.2163 | No |
| 13 | HLA-DQB1 | na | 13160 | 0.045 | -0.2155 | No |
| 14 | C7 | na | 13878 | 0.037 | -0.2333 | No |
| 15 | HLA-DOA | na | 16250 | 0.012 | -0.3007 | No |
| 16 | C8B | na | 17141 | 0.003 | -0.3261 | No |
| 17 | SSB | na | 17878 | -0.005 | -0.3469 | No |
| 18 | SNRPD1 | na | 18985 | -0.017 | -0.3775 | No |
| 19 | HLA-DRB1 | na | 19037 | -0.017 | -0.3776 | No |
| 20 | HLA-DMA | na | 19878 | -0.027 | -0.3997 | No |
| 21 | C1QC | na | 20504 | -0.034 | -0.4150 | No |
| 22 | FCGR2B | na | 21581 | -0.047 | -0.4423 | No |
| 23 | CTSG | na | 22930 | -0.064 | -0.4761 | No |
| 24 | SNRPB | na | 23129 | -0.067 | -0.4764 | No |
| 25 | HLA-DOB | na | 23582 | -0.074 | -0.4835 | No |
| 26 | GRIN2B | na | 24135 | -0.083 | -0.4929 | No |
| 27 | HLA-DRB4 | na | 24359 | -0.087 | -0.4924 | No |
| 28 | HLA-DRB3 | na | 24377 | -0.087 | -0.4860 | No |
| 29 | SNRPD3 | na | 24607 | -0.092 | -0.4853 | No |
| 30 | CD80 | na | 26418 | -0.126 | -0.5275 | No |
| 31 | C1QA | na | 26646 | -0.131 | -0.5236 | No |
| 32 | TRIM21 | na | 26659 | -0.132 | -0.5135 | No |
| 33 | ACTN1 | na | 28221 | -0.171 | -0.5450 | No |
| 34 | IL10 | na | 28370 | -0.176 | -0.5352 | No |
| 35 | FCGR3A | na | 29886 | -0.221 | -0.5613 | Yes |
| 36 | CD40LG | na | 30278 | -0.236 | -0.5539 | Yes |
| 37 | ACTN2 | na | 30345 | -0.239 | -0.5368 | Yes |
| 38 | HLA-DRA | na | 30472 | -0.244 | -0.5210 | Yes |
| 39 | HLA-DPA1 | na | 30909 | -0.263 | -0.5126 | Yes |
| 40 | C4B | na | 30983 | -0.267 | -0.4934 | Yes |
| 41 | FCGR2C | na | 31076 | -0.272 | -0.4744 | Yes |
| 42 | TNF | na | 31738 | -0.308 | -0.4689 | Yes |
| 43 | HLA-DMB | na | 32287 | -0.339 | -0.4577 | Yes |
| 44 | C5 | na | 32411 | -0.348 | -0.4336 | Yes |
| 45 | CD40 | na | 32420 | -0.349 | -0.4060 | Yes |
| 46 | C4A | na | 32536 | -0.357 | -0.3810 | Yes |
| 47 | IFNG | na | 32943 | -0.389 | -0.3617 | Yes |
| 48 | ACTN3 | na | 33200 | -0.413 | -0.3362 | Yes |
| 49 | C1R | na | 33226 | -0.416 | -0.3038 | Yes |
| 50 | C2 | na | 33722 | -0.482 | -0.2798 | Yes |
| 51 | C1QB | na | 33797 | -0.494 | -0.2426 | Yes |
| 52 | HLA-DPB1 | na | 34158 | -0.570 | -0.2077 | Yes |
| 53 | C3 | na | 34350 | -0.633 | -0.1628 | Yes |
| 54 | C1S | na | 34370 | -0.640 | -0.1125 | Yes |
| 55 | HLA-DQA2 | na | 34566 | -0.745 | -0.0589 | Yes |
| 56 | HLA-DQA1 | na | 34604 | -0.798 | 0.0036 | Yes |
Table: GSEA details [plain text format]

  

Fig 2: KEGG\_SYSTEMIC\_LUPUS\_ERYTHEMATOSUS      
 Blue-Pink O' Gram in the Space of the Analyzed GeneSet

  

Fig 3: KEGG\_SYSTEMIC\_LUPUS\_ERYTHEMATOSUS: Random ES distribution      
 Gene set null distribution of ES for **KEGG\_SYSTEMIC\_LUPUS\_ERYTHEMATOSUS**
